# Daurisoline Inhibits ESCC by Inducing G1 Cell Cycle Arrest and Activating ER Stress to Trigger Noxa-Dependent Intrinsic and CHOP-DR5-Dependent Extrinsic Apoptosis via p-eIF2*α*-ATF4 Axis

**DOI:** 10.1155/2022/5382263

**Published:** 2022-08-04

**Authors:** Shuying Yuan, Yongfu Pan, Tong Xu, Li Zhang, Xihui Chen, Fengying Wang, Qian Liu, Lijun Jia

**Affiliations:** Cancer Institute of Traditional Chinese Medicine, Longhua Hospital, Shanghai University of Traditional Chinese Medicine, Shanghai, China

## Abstract

Esophageal squamous cell carcinoma (ESCC), one of the most malignant human cancers in clinic, requires novel treatment. Daurisoline (DAS) is a component of traditional Chinese herb, which exhibits anti-cancer effects by autophagy inhibition and metastasis suppression. However, the effect and mechanism of DAS on ESCC remain unclear. Here, we found that DAS inhibited cell proliferation and colony formation in both human ESCC cell lines EC1 and ECA109. Mechanistically, DAS induced p21-/p27-dependent G1 phase cell cycle arrest and apoptosis in a dose-dependent manner. The induction of apoptosis by DAS was largely dependent on the activation of the transcription factor ATF4 and its downstream NOXA-dependent intrinsic and CHOP-DR5-dependent extrinsic apoptotic pathway. ATF4 activation induced by DAS was due to the generation of excessive reactive oxygen species (ROS) and the subsequent activation of endoplasmic reticulum (ER) stress through the p-eIF2*α*-ATF4 signal pathway, which can be largely abrogated by N-acetylcysteine (NAC), a scavenger of ROS. Moreover, DAS treatment significantly inhibited tumor growth and reduced tumor weight in the tumor xenograft mouse model by up-regulating key proteins related to cell cycle arrest and apoptotic pathway. Taken together, these findings identified DAS as a novel candidate for the treatment of ESCC.

## 1. Introduction

According to Global Cancer Statistics 2020, esophageal cancer is one of the most lethal cancers in the world, which ranks seventh in terms of incidence and sixth in mortality overall. Esophageal squamous cell carcinoma (ESCC) is the most common histology subtype of esophageal cancer, which has a high incidence in China [1]. Surgical resection, chemoradiotherapy, and targeted therapy are the primary clinical treatments. However, survival improvement was minimal due to the limited efficacy and severe adverse effects in practice [2]. Chinese herbal medicines as an adjuvant during cancer therapy possess great advantages, including but not limited to suppressing tumor progression, increasing the sensitivity of radio-, chemo-, or targeted therapeutics, reducing adverse reactions, and improving immunity [3].

**Figure 1:**
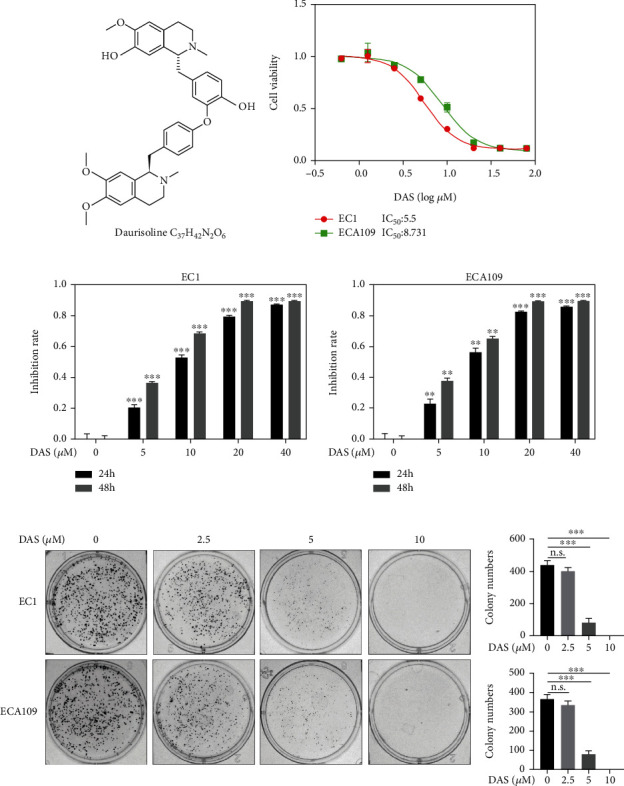
Daurisoline inhibited the viability of ESCC cells. (a) The chemical structure of DAS. (b) Human ESCC cell lines EC1 and ECA109 were treated with indicated concentrations of DAS for 72 hours, and the IC_50_ was determined by CCK-8 assay. (c) EC1 and ECA109 cell lines were treated with indicated concentrations of DAS for 24 and 48 hours. (d) Representative images of three independent experiments were shown for the inhibition of colony formation by DAS and a statistical graph of the relative number of colonies formed.

Daurisoline (DAS) is the main effective component extracted from the traditional Chinese herbal Rhizoma Menispermi (Figure 1(a)) [4]. Previous studies have shown that DAS has a wide range of cardiovascular and cerebrovascular pharmacological effects, including multiple modulations of focal ischemia/reperfusion injury, arrhythmia, platelet aggregation, antagonizing potassium, and calcium channel [5–8]. Moreover, DAS exhibits anti-cancer effects through inhibiting autophagy or vasculogenic mimicry (VM) formation. DAS has been reported as an inhibitor of autophagy by disturbing the autophagosome formation and fusion process and impairing lysosomal function, which remarkably enhances the efficiency of photothermal cancer therapy [9] and promotes the sensitivity of chemotherapeutic drugs such as cisplatin, camptothecin, and sorafenib [10–12]. In addition, DAS inhibited tumor invasion and metastasis via destabilizing *β*-catenin or repressed VM formation by inactivating RhoA/ROCK2-mediated AKT or ERK-p38 MAPK signaling pathway [12, 13]. Recently, a few studies have emphasized that DAS could induce apoptosis of cancer cells [10, 12]. However, the underlying molecular mechanism of apoptosis and its upstream activation events remain unclear.

In response to stimuli, cells may die from accidental cell death (ACD), a biologically uncontrolled process, or regulated cell death (RCD) including apoptosis [14]. Apoptosis consists of two major subtypes, namely, intrinsic and extrinsic apoptosis. Intrinsic apoptosis is ignited by mitochondrial outer membrane permeabilization (MOMP), which is tightly controlled by the BCL-2 family that leads to the release of mitochondrial proteins and subsequent activation of initiator caspase 9. While active caspase 8 and caspase 10 are the main effectors of death receptor-mediated extrinsic apoptosis [15]. Apoptosis is activated by a set of stimuli, including DNA damage, metabolic stress, and ER stress [16].

Short-time ER stress may be beneficial for cells, while long-term or severe ER stress is lethal [17, 18]. Once ER stress is initiated, three ER stress sensors including PKR-like ER kinase (PERK), inositol-requiring enzyme 1 (IRE1), and activating transcription factor 6 (ATF6) would detect the accumulation of unfolded or misfolded proteins at the onset of ER stress and trigger the unfolded protein response (UPR) [19]. PERK is a major transducer of the ER stress response and directly phosphorylates *α*-subunit of eukaryotic initiation factor 2 (eIF2*α*), which inhibits global protein translation. However, phosphorylated eIF2*α* (p-eIF2*α*) promotes the translation of activating transcription factor 4 (ATF4) to regulate the expression of some molecules involved in protein synthesis, amino acid transport, secretion, and even apoptosis [20, 21].

In this study, it was the first time to explore the anti-cancer effect and mechanism of DAS in ESCC both *in vitro* and *in vivo*. On the molecular level, DAS inhibited ESCC cell proliferation by inducing p21-/p27-dependent G1 cell cycle arrest. Moreover, Noxa-dependent intrinsic and CHOP-DR5-dependent extrinsic apoptosis was significantly triggered by DAS via p-eIF2*α*-ATF4 axis, which was due to excessive ROS production and the subsequent activation of ER stress. In summary, these findings indicated that DAS might be a promising reagent for ESCC treatment.

## 2. Materials and Methods

### 2.1. Cell Culture

Human ESCC cell lines EC1 and ECA109 were provided by the Type Culture Collection of the Chinese Academy of Sciences (Shanghai, China). All cells were cultured in Dulbecco's Modified Eagle's Medium (DMEM, cyclone, Logan, UT), containing 10% fetal bovine serum (FBS, Biochrom AG, Berlin, Germany) and 1% penicillin-streptomycin solution (Gibco, USA), at 37°C in a humidified atmosphere with 5% CO_2_.

### 2.2. Reagents

Daurisoline (DAS) was purchased from MCE (MedChemExpress, Shanghai, China). DAS was dissolved in dimethyl sulfoxide (DMSO, Sigma-Aldrich, Germany) and stored at −80°C for the *in vitro* study. For the *in vivo* study, DAS was firstly dissolved in 50% PEG300 and then in 50% saline (MCE, Shanghai, China). Z-VAD-FMK (Z-VAD) was purchased from MCE. Necrostatin-1 (Nec-1), necrosulfonamide (NSA), and ferrostatin-1 (Fer-1) were purchased from Selleck (Selleck, Shanghai, China). N-acetylcysteine (NAC) was purchased from MCE.

### 2.3. Cell Viability Assay and Colony Formation Assay

Cell viability was evaluated using cell counting kit-8 (CCK-8, ShareBio, Shanghai, China) and ATPlite (BD Pharmingen, Franklin Lakes, New Jersey, United States) following the manufacturer's protocol. Briefly, cells were seeded in 96-well plates (2 × 10^3^ cells/well) in triplicate and cultured overnight. On the following day, the cells were treated as described in each experiment and then incubated at 37°C for a designated time, and the control was treated with 0.1% DMSO. Next, the cell viability was measured by a microplate reader (BIOTEK Synergy HT, USA).

For colony formation assay, cells were plated into six-well plates (500 cells per well) in triplicate and allowed to adhere overnight, then treated with the indicated concentrations of DAS and cultured for 10 days. Finally, cells were fixed with 4% paraformaldehyde for 30 minutes and stained with 0.1% crystal violet. Colonies with more than 50 cells each were counted and photographed with a gel imager (GelDoc XR System, Bio-rad, USA).

### 2.4. Cell Cycle Analysis

Cells were harvested after treatment with DAS for 24 hours and then fixed in 70% ethanol at -20°C overnight and stained with propidium iodide (PI, ShareBio, Shanghai, China) for 30 minutes in a PI buffer solution at room temperature in the dark, and stained cells were analyzed with flow cytometry (Beckman Coulter Fullerton, CA, USA). Data were analyzed by FlowJo 7.6 software.

### 2.5. Apoptosis Assay

Cells were harvested and washed with cold PBS after treatment with DAS for 24 hours and then incubated with Annexin V-fluorescein isothiocyanate and propidium iodide (Annexin V-FITC/PI, ShareBio, Shanghai, China) for 30 minutes in a dark room. Following, apoptotic cells were analyzed using flow cytometry (Beckman Coulter, Fullerton, CA, USA).

### 2.6. Western Blot Analysis

After various treatments as indicated, cancer cells were harvested, and the total protein was lysed in radio immunoprecipitation assay (RIPA) lysis buffer (Beyotime, Shanghai, China) and then quantified by a BCA kit (Epizyme, Shanghai, China). Equal amounts of proteins were resolved on 10%-12.5% SDS-PAGE gels, followed by transferring the proteins to an Immobilon PVDF Membrane (Merck Millipore Ltd, Tullagreen, Ireland). PVDF membranes with proteins were blocked with 5% skim milk for 1 hour, incubated with primary antibodies overnight at 4°C, and then conjugated with secondary antibodies (Cell Signaling Technology, Danvers, MA, USA) at room temperature for 1 hour. Signals were visualized by ECL reagent (Epizyme, Shanghai, China) and photographed by Tanon 5200 visualizer (Tanon, Shanghai, China).

The primary antibodies used are as follows: p27, p21, Cyclin D1, PARP, cleaved PARP (c-PARP), cleaved caspase 3, cleaved caspase 9, cleaved caspase 7, cleaved caspase 8, Noxa, Bak, Bax, Bik, Bim, Bad, Puma, Bcl2, Bcl-xl, Mcl-1, XIAP, BID, TRAIL, DR4, DR5, CHOP, ATF4, eIF2*α*, and p-eIF2*α* were from Cell Signaling Technology (USA). Cyclin E, CDK2, CDK4, CDK6, Fas, DR3, c-Myc, and p53 were from Santa Cruz Biotechnology (USA). TNFR1 and TNFR2 were from Proteintech (USA). *β*-Actin was from HuaBio (China).

### 2.7. Quantitative Real-Time PCR (Q-PCR)

Total RNA was isolated using the Ultrapure RNA Kit (Com Win Biotech, Beijing, China) following the manufacturer's protocol. Then, the PrimerScript reverse transcription reagent kit (Vazyme Biotech, Nanjing, China) was used to reverse total RNA to cDNA. The primers used for real-time PCR were summarized in Table 1. Reactions were performed using the Power SYBR Green PCR MasterMix (Vazyme Biotech, Nanjing, China) on the ABI 7500 thermocycler (Applied Biosystems, Foster City, CA, United States) following the instrument instructions. As an internal control, *β*-actin levels were quantified in parallel with target genes. Normalization and fold change for each of the genes were calculated using the 2^-∆∆CT^ method.

### 2.8. RNA Interfering (RNAi)

Cells were transfected with siRNA oligonucleotides by using Lipofectamine 2000 (Invitrogen, USA), Opti-MEM (Invitrogen, USA) was used to incubate with siRNA and Lipofectamine 2000 separately for 5 minutes at room temperature and mixed for 15 minutes, and then, the mixture together with the serum-free medium was applied to the cells. The sequences of siRNA were synthesized by GenePharma (Shanghai, China) and summarized in Table 2.

### 2.9. Measurement of ROS Generation

The intracellular ROS production was monitored by cell-permeable ROS indicator 2′,7′-dichlorodihydrofluorescein diacetate (DCFH-DA) (Sigma-Aldrich, Germany). DAS-treated ESCC cells were stained with 10 *μ*M DCFDA for 30 minutes at 37°C in the darkroom. Next, the level of ROS generation was detected with flow cytometry (Beckman Coulter, Fullerton, CA, USA). Data were analyzed by FlowJo 7.6 software.

### 2.10. Establishment of Tumor Xenograft Mouse Model

The mouse experiments were conducted in a pathogen-free environment in the animal facility of Longhua Hospital. ECA109 cells (1.5 × 10^6^) were suspended in 50 *μ*l PBS and 50 *μ*l matrigel (BD, USA) and injected subcutaneously into the bilateral buttocks of 5- to 6-week-old female BALB/c nude mice (Lingchang Biological Technology, Shanghai, China). Two days later, the tumor cell-inoculated mice were randomly divided into control and DAS-treatment groups (six mice per group). Each mouse was treated with either PEG300 (control) or DAS (40 mg/kg) via oral gavage once a day for 13 consecutive days. Tumor size was measured every day by a digital caliper and calculated as *V* = *L*∗*S*^2^/2 (*L* is the longest diameter, and *S* is the shortest diameter). The mice's weights were measured with an electronic scale every other day. After the treatment, mice were sacrificed; tumors were excised, weighed, and photographed; and tissues were frozen and preserved at -80°C for the following experiment analysis. Liver and kidney tissues of mice were fixed in 4% paraformaldehyde and stained with H&E. In addition, the serum of mice was collected to detect the liver and kidney function. All experiments were performed according to a protocol approved by the Experimental Animal Ethical Committee of the Longhua Hospital affiliated with Shanghai University of Chinese Medicine. The number of the ethical approval is LHERAW-22050.

### 2.11. Immunohistochemical Staining

The mouse tumor tissue sections were stained by immunohistochemistry (IHC) with a Ki-67 antibody. The tissue sections were dehydrated and subjected to peroxidase blocking. Primary antibodies were added and incubated at 37°C for 1 hour, a biotinylated secondary antibody was used for 30 minutes, 3′-diaminobenzidine (DAB) was put into use as a chromogen substrate, and Harris's hematoxylin was used as a counterstain. Images were acquired with a digital slice scanner (Konfoong biotech international, Ningbo, China). Immunoreactivity values in tumor tissues were scored according to cell staining intensity and percentage score of positive cells, for example, negative-no positive staining (0), weak positive-light yellow (1), positive-brown yellow (2), and strong positive-tan (3). The percentage score of positive cells are ≤25% (1), 26-50% (2), 51-75% (3), and >75% (4). Finally, the two scores are multiplied to obtain the final score [22].

### 2.12. Statistical Analysis

All experiments were carried out at least three times, and data were displayed as mean ± standard deviation. The statistical significance of differences between groups was assessed using GraphPad Prism 9 software (GraphPad Software, Inc, San Diego, CA, USA). The Student's *t*-test was used for the comparison of parameters between two groups. Four levels of significance were used for all tests (^∗^*p* < 0.05, ^∗∗^*p* < 0.01, and ^∗∗∗^*p* < 0.001, n.s. denotes no significance).

## 3. Results

### 3.1. Daurisoline Inhibited the Viability of ESCC Cells

To determine whether DAS is an effective agent against ESCC, we carried out CCK-8 assay and found that the IC_50_ values of DAS for EC1 and ECA109 were 5.50 *μ*M and 8.73 *μ*M, respectively, and the inhibitory effect was positively correlated with time and concentration (Figures 1(b) and 1(c)). Furthermore, these observations were confirmed by colony formation assay, and 5 *μ*M DAS treatment significantly reduced clone numbers in comparison to the control group in both cell lines (Figure 1(d)). Taken together, these data indicated that DAS was a potent inhibitor for the proliferation of ESCC cells.

### 3.2. Daurisoline Induced p21-/p27-Dependent G1 Phase Cell Cycle Arrest

**Figure 2:**
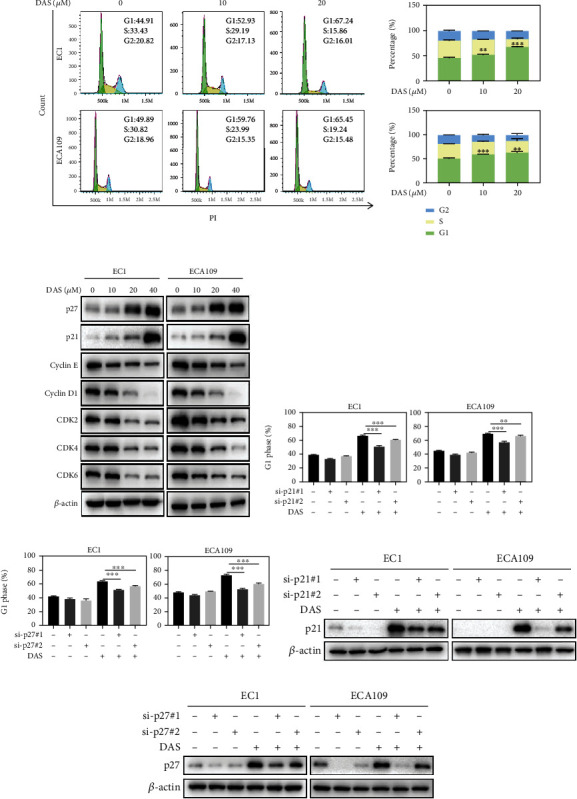
Daurisoline induced p21-/p27-dependent G1 phase cell cycle arrest. (a) EC1 and ECA109 cells were incubated with DMSO or DAS for 24 hours, followed by PI staining and FACS analysis for cell cycle profiling. The percentage of each cell cycle was counted to draw a statistical graph. (b) EC1 and ECA109 cells were incubated with DAS at indicated concentrations, and proteins were extracted and detected by western blot. (c-f) EC1 and ECA109 cells were transfected with control or two siRNA sequences of p21 or p27, then treated with DAS (20 *μ*M) for 24 hours, and finally subjected to PI staining and FACS analysis. The protein levels of p21 or p27 were determined by western blot.

To further explore the inhibitory mechanism of DAS, PI staining and flow cytometry were used to analyze the cell cycle. As shown in Figure 2(a), the ratio of G1 phase was enhanced by DAS in a dose-dependent manner. Moreover, the critical regulators of G1 phase, cyclin-CDK inhibitor proteins p21 and p27, were strongly up-regulated, while their counteraction complexes CDK2-Cyclin E and CDK4/6-Cyclin D1 were sharply down-regulated under the treatment of DAS, indicating that DAS triggered G1 cell cycle arrest (Figure 2(b)). In addition, knockdown of p21 or p27 by siRNA partially reversed DAS-induced G1 cell cycle arrest (Figures 2(c)–2(f)). These data highlighted that DAS suppressed the growth of ESCC cells via p21-/p27-dependent G1 phase cell cycle arrest.

### 3.3. Daurisoline Induced Cell Death Mainly through Caspase-Dependent Apoptosis

**Figure 3:**
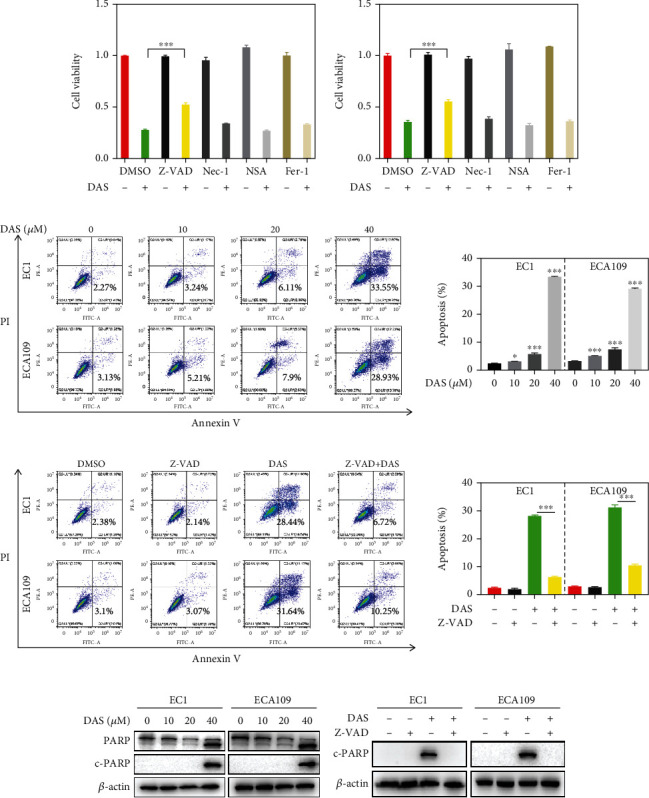
Daurisoline induced cell death mainly through caspase-dependent apoptosis. (a) EC1 and ECA109 cells were treated with Z-VAD (20 *μ*M), Nec-1 (50 *μ*M), NSA (10 *μ*M), and Fer-1 (1 *μ*M) or combined with DAS (40 *μ*M) for 24 hours, followed by the ATPlite assay. (b, c) EC1 and ECA109 cells were treated with indicated concentrations of DAS or DAS (40 *μ*M) and Z-VAD (20 *μ*M) for 24 hours, then stained with an Annexin-V-FITC apoptosis detection kit and analyzed with FCAS, the proportion of apoptotic cells was counted, and the statistical graph was made. (d, e) EC1 and ECA109 cells were treated with indicated concentrations of DAS or DAS (40 *μ*M) and Z-VAD (20 *μ*M) for 24 hours, and proteins were extracted and detected by western blot.

During the experiment, obvious cell death was observed when cells were exposed to DAS under the microscope (data not shown), suggesting that DAS might induce cell death. To explore the predominant form of cell death, apoptosis inhibitor Z-VAD-FMK (Z-VAD), necroptosis inhibitor necrostatin-1 (Nec-1), necrosulfonamide (NSA), and ferroptosis inhibitor ferrostatin-1 (Fer-1) were incubated with DAS to treat EC1 and ECA109 cells. Only Z-VAD, the inhibitor of caspase, could significantly rescue the DAS-induced cell death, suggesting that apoptosis may contribute the most (Figure 3(a)). Additionally, the proportion of apoptotic cells and the marker of apoptosis, cleaved-PARP were enhanced in a dose-dependent manner, supporting that DAS induced apoptosis in ESSC cells. Strikingly, these phenomena can be largely abrogated when DAS was treated with Z-VAD together (Figures 3(b)–3(e)). These results strongly demonstrated that DAS induced cell death mainly through caspase-dependent apoptosis.

### 3.4. Daurisoline Triggered Noxa-Dependent Intrinsic Apoptosis

**Figure 4:**
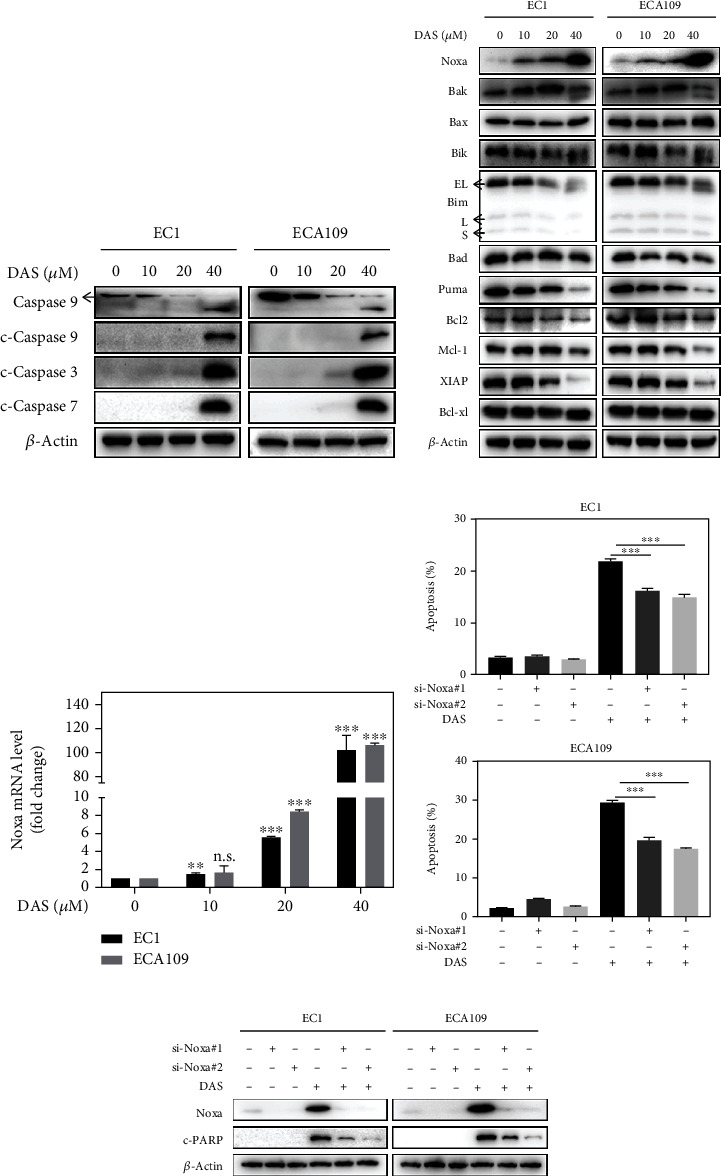
Daurisoline triggered Noxa-dependent intrinsic apoptosis. (a) EC1 and ECA109 cells were treated with DAS as described above and were subjected to western blot. (b) EC1 and ECA109 cells were incubated with DAS at indicated concentrations, and proteins were extracted and detected by western blot (EL: 23 kDa; L: 15 kDa; S: 12 kDa). (c) The mRNA level of Noxa was quantified by Q-PCR. (d, e) EC1 and ECA109 cells were transfected with control or two siRNA sequences of Noxa, then treated with DAS (30 *μ*M) for 24 hours. Apoptosis induction was quantified by Annexin V-FITC/PI double-staining analysis. Noxa and cleaved PARP protein levels were detected by western blot.

To further characterize the underlying molecular mechanism of apoptosis, classical hallmark proteins related to intrinsic apoptosis were primarily detected. As shown in Figure 4(a), DAS induced obvious up-regulation of cleaved caspase 9, cleaved caspase 3, and cleaved caspase 7 in EC1 and ECA109 cells, suggesting that DAS triggered the intrinsic apoptosis. Intrinsic apoptosis is tightly controlled by the BCL-2 family, which is composed of anti-apoptotic and pro-apoptotic proteins. During DAS treatment, almost anti-apoptotic proteins Bcl2, Mcl-1, and XIAP were down-regulated except for Bcl-xl, while none of pro-apoptotic proteins except Noxa was found to be stimulated in both mRNA and protein levels (Figures 4(b) and 4(c)), which supports a predominant role of Noxa in this event. To further verify the indispensable role of Noxa in DAS-induced intrinsic apoptosis, depletion of Noxa by specific siRNA significantly inhibited apoptosis induced by DAS (Figures 4(d) and 4(e)). These results elucidated that DAS transactivated Noxa and triggered the intrinsic apoptosis in ESCC cells.

### 3.5. Daurisoline Triggered CHOP-DR5-Dependent Extrinsic Apoptosis

**Figure 5:**
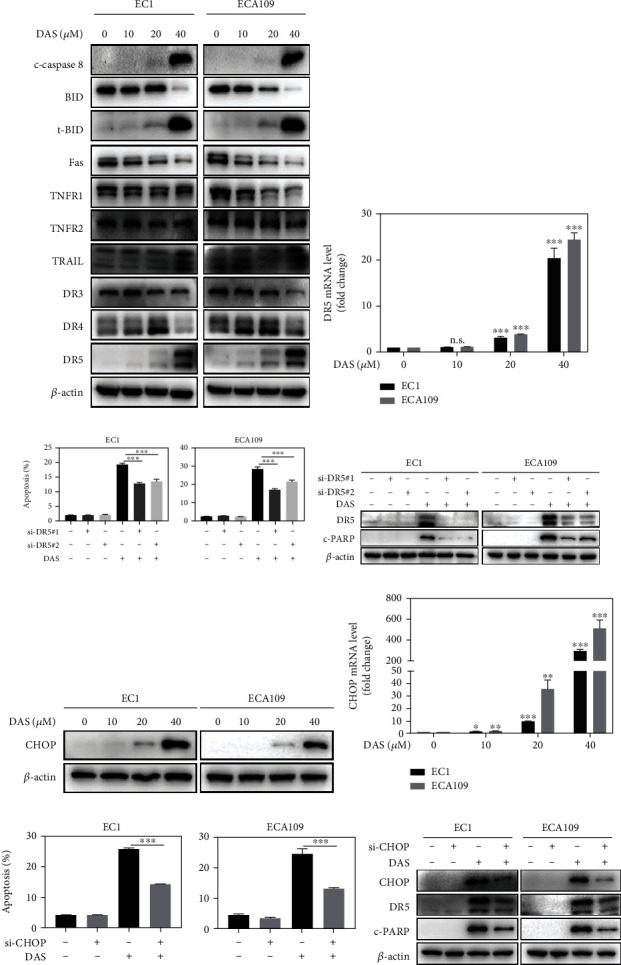
Daurisoline triggered CHOP-DR5 dependent extrinsic apoptosis. (a) EC1 and ECA109 cells were treated with DAS as described above and were subjected to western blot. (b) The mRNA level of DR5 was quantified by Q-PCR. (c, d) EC1 and ECA109 cells were transfected with control or two siRNA sequences of DR5, then treated with DAS (30 *μ*M) for 24 hours. Apoptosis induction was quantified by Annexin V-FITC/PI double-staining and FACS analysis. DR5 and cleaved PARP protein levels were quantified by western blot. (e, f) EC1 and ECA109 cells were treated with indicated concentrations of DAS for 24 hours, and proteins were extracted and detected by western blot. The mRNA level of CHOP was quantified by Q-PCR. (g, h) EC1 and ECA109 cells were transfected with control or CHOP siRNA, then treated with DAS (30 *μ*M) for 24 hours. Apoptosis induction was quantified by Annexin V-FITC/PI double-staining and FACS analysis. Proteins were extracted and detected by western blot.

To explore whether DAS can induce extrinsic apoptosis, we carried out western blot analysis and found that both the extrinsic apoptosis-related marker cleaved caspase 8 and its downstream-mediated truncated BID (t-BID) were up-regulated by DAS, suggesting that DAS can further trigger intrinsic apoptosis. The activation of extrinsic apoptosis is governed by a series of cell death receptors and ligands. Among all proteins, only DR5 was significantly up-regulated in both mRNA and protein levels (Figures 5(a) and 5(b)), suggesting that DR5 was important for DAS-induced extrinsic apoptosis. Indeed, knockdown of DR5 partially reversed DAS-induced apoptosis (Figures 5(c) and 5(d)). Given that CHOP is known to be critically involved in the transcription of the DR5 gene [23], we next examined whether DAS-induced DR5 up-regulation was related to CHOP. Consistent with the results of DR5, both the protein and mRNA levels of CHOP were up-regulated by DAS (Figures 5(e) and 5(f)). And knockdown of CHOP rescued the up-regulation of DR5 and cleaved PARP, as well as apoptosis, which confirmed the direct involvement of CHOP in the DAS-mediated transcriptional activation of DR5 (Figures 5(g) and 5(h)). These results highlighted the key role of CHOP-DR5 in extrinsic apoptosis triggered by DAS.

### 3.6. ATF4 Was Responsible for Daurisoline-Induced Intrinsic and Extrinsic Apoptosis

**Figure 6:**
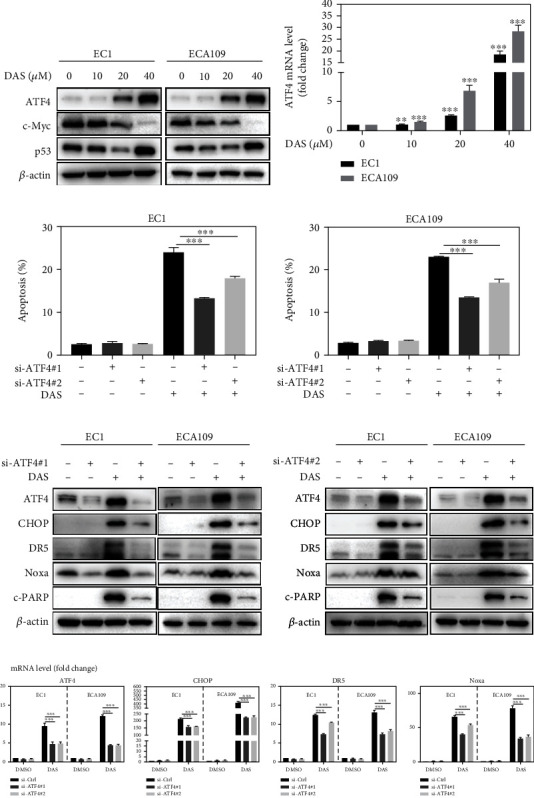
ATF4 was responsible for daurisoline-induced intrinsic and extrinsic apoptosis. (a) EC1 and ECA109 cells were treated with DAS as described above and were subjected to western blot. (b) The mRNA level of ATF4 was quantified by Q-PCR. (c-e) EC1 and ECA109 cells were transfected with control or two siRNA sequences of ATF4, then treated with DAS (30 *μ*M) for 24 hours. Apoptosis induction was quantified by Annexin V-FITC/PI double-staining and FACS analysis. Proteins were extracted and detected by western blot. The mRNA level of ATF4, CHOP, DR5, and Noxa was quantified by Q-PCR.

Previous studies reported that ATF4 is not only the classical upstream signal of CHOP but also the transcription activator of Noxa [24–26]. Thus, we tested the expression level of ATF4 protein and other potential transcriptional regulators, including c-Myc and p53, and found that ATF4 protein could be up-regulated by DAS in a dose-dependent manner, as well as the mRNA level (Figures 6(a) and 6(b)). Knockdown of ATF4 by siRNA largely reversed the apoptosis induced by DAS (Figure 6(c)). Simultaneously, down-regulation of ATF4 significantly inhibited the induction of CHOP, DR5, and Noxa by DAS at both protein and mRNA levels in EC1 and ECA109 cells (Figures 6(d) and 6(e)). Collectively, these results indicated that ATF4 is an essential transcription factor in DAS-induced Noxa-dependent intrinsic and CHOP-DR5-dependent extrinsic apoptosis.

### 3.7. ROS-Dependent Activation of the p-eIF2*α*-ATF4 Axis Was Essential for Daurisoline-Induced Apoptosis

**Figure 7:**
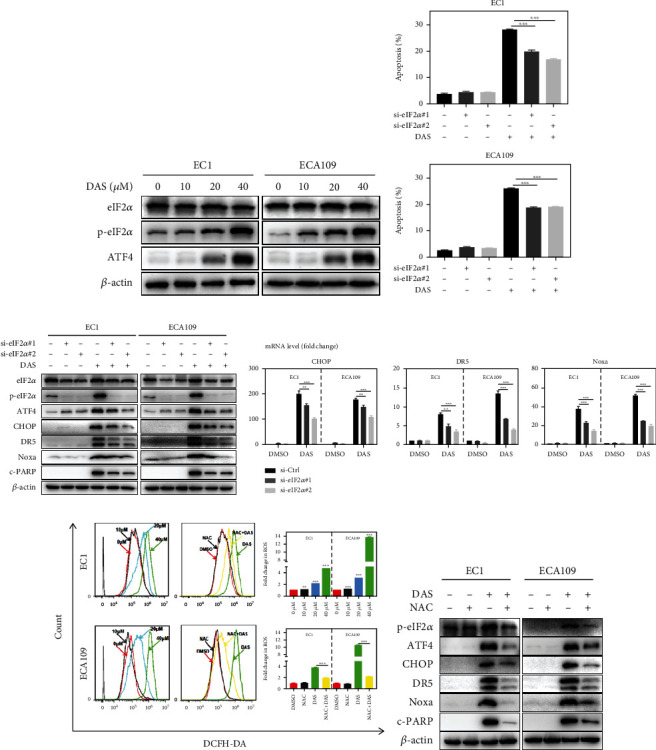
ROS-dependent activation of the p-eIF2*α*-ATF4 axis was essential for daurisoline-induced apoptosis. (a) EC1 and ECA109 cells were treated with DAS as described above, and proteins were subjected to western blot. (b-d) EC1 and ECA109 cells were transfected with control or two siRNA sequences of eIF2*α*, then treated with DAS (30 *μ*M) for 24 hours. Apoptosis induction was quantified by Annexin V-FITC/PI double-staining and FACS analysis. Proteins were extracted and detected by western blot. The mRNA level of CHOP, DR5, and Noxa was quantified by Q-PCR. (e, f) EC1 and ECA109 cells were treated with indicated concentrations of DAS or DAS (40 *μ*M) and NAC (10 mM) for 24 hours, and ROS generation was determined by DCFH-DA staining and FACS analysis. Proteins were extracted and detected by western blot.

Considering the essential role of ATF4 in cellular apoptosis triggered by DAS, we wondered that ER stress may be involved in the regulation of ATF4 [27]. As shown in Figure 7(a), ATF4 and p-eIF2*α* were elevated in a dose-dependent manner after DAS treatment, while no significant change was observed in eIF2*α*, suggesting that ER stress was induced by DAS. To further verify the role of eIF2*α* on DAS-induced apoptosis, specific siRNA was used to silence eIF2*α*. As expected, depletion of eIF2*α* significantly rescued DAS-induced apoptosis (Figure 7(b)), and the result was similar to ATF4 depletion during DAS treatment. Moreover, eIF2*α* depletion attenuated the up-regulation of p-eIF2*α*, ATF4, CHOP, DR5, Noxa, and cleaved PARP induced by DAS (Figure 7(c)), as well as the mRNA level of CHOP, DR5, and Noxa (Figure 7(d)). These data indicated that DAS-induced apoptosis was due to the initiation of ER stress via the p-eIF2*α*-ATF4 axis.

ER stress is an essential response to excessive ROS generation [28]. Through DCFH-DA staining, we found that DAS induced ROS production in a dose-dependent manner in both EC1 and ECA109 cells. In addition, pre-treatment of NAC, a ROS scavenger, greatly reversed the ROS level and the up-regulation of p-eIF2*α*, ATF4, CHOP, DR5, Noxa, and cleaved PARP induced by DAS (Figures 7(e) and 7(f)). Taken together, these findings indicated that DAS-induced apoptosis was due to ROS production, which triggered ER stress via the p-eIF2*α*-ATF4 axis.

### 3.8. Daurisoline Suppressed the Growth of ESCC *In Vivo*

**Figure 8:**
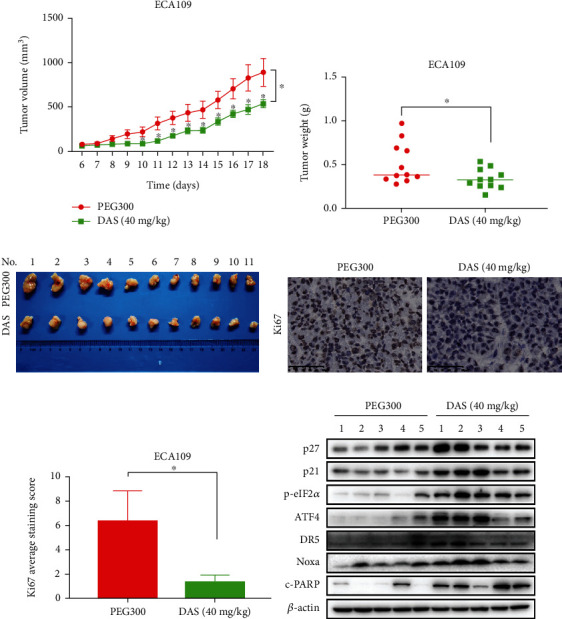
Daurisoline suppressed the growth of ESSC *in vivo*. (a) Nude mice were subcutaneously transplanted with ECA109 cells and treated with DAS as indicated in materials and methods. Tumor size was determined with a caliper every day, and the volume was calculated to construct a growth curve. (b) The tumor weight was measured with an electronic scale. (c) Mice were sacrificed, and tumor tissues were harvested and photographed. (d) IHC staining for Ki-67 in tumor sections. (e) A statistical graph of the Ki-67 average staining score of the PEG300 control group and DAS (40 mg/kg) treatment group. (f) Tissue proteins were extracted and detected by western blot.

To further validate the anti-cancer effect of DAS *in vivo*, the tumor xenograft mice model was established as described. DAS treatment significantly inhibited tumor growth and reduced tumor weight compared to the control group (Figures 8(a)–8(c)). Moreover, DAS significantly inhibited the expression of Ki-67 by immunohistochemical analysis (Figures 8(d) and 8(e)), which emphasized the inhibitory effect of DAS in ESCC. Significantly, western blot analysis of tumor tissue confirmed that DAS markedly up-regulated the expression levels of p27, p21, p-eIF2*α*, ATF4, DR5, Noxa, and cleaved PARP compared with control (Figure 8(f)), which was consistent with *in vitro* experiments. In addition, there was no significant difference in body weight during the whole experiment (Figure Supplement 1A). Histological and serum examination of the liver and kidney did not reveal any overt changes (Figures Supplement 1B-C), which verified the safety of DAS *in vivo*. Taken together, these results demonstrated that DAS is a potential anti-cancer reagent with strong treatment efficacy and safety.

## 4. Discussion

Esophageal cancer is one of the lethal factors of human health, and it is urgent to identify novel targets and develop more effective drugs. Natural products with unique structures and mechanisms of action may generate chemical affinity that interacts with most or possibly therapeutic targets and play an essential role in cancer therapy. Representative taxol, vinblastine, and camptothecin are already adopted in clinic, which has remarkable treatment efficacy and low toxicity [29]. Previous studies revealed that the natural product daurisoline has remarkable anti-tumor activity, while there was no research of DAS on esophageal cancer. Through a systematic and comprehensive study, we demonstrated that daurisoline exhibited a significant inhibitory effect on the growth of ESCC cells *in vitro* and *in vivo* through inducing G1 cell cycle arrest and apoptosis.

Accelerated cell cycle progression contributes to the continuous proliferation and rapid growth of cancer cells, and the progression of cell cycle is regulated by the coordinated activities of cyclin/cyclin-dependent kinases (CDKs) complexes [30]. Besides, CDK activity is regulated by INK4 protein and the Cip and Kip family (including p21, p27, and p57 members) [31]. While CDKs are often over-expressed, inhibitory proteins like p21 and p27 are generally down-regulated in human cancer [32]. Undoubtedly, selective CDK inhibitors may provide therapeutic benefits for halting tumor growth [30]. Molecularly, we found that the DAS treatment mainly induced cell cycle arrest at the G1 phase, which depends on p21 and p27 up-regulation to inhibit the cyclin/CDK complex's activity. Rescue experiments further revealed that additional p21 or p27 knockdown partly reversed the DAS-induced G1 cell cycle arrest. However, the potential regulatory factor of p21 and p27 in DAS-elicited cell cycle inhibition was unclear, and future studies will be performed to elucidate the mechanism of how DAS up-regulated p21 or p27 in ESCC cells.

In response to ROS-mediated oxidative stress, accumulation of unfolded or misfolded proteins triggers a cellular adaptive procedure known as ER stress, and quite a few studies indicated the regulation of ROS on ER stress activation in a variety of cancer or normal cell lines [28, 33–35]. Proper functioning of ER stress is critical to many aspects of cellular physiology, and appropriate ER stress is beneficial to maintaining intracellular homeostasis. However, persistent or intense ER stress will trigger programmed cell death, even apoptosis [36, 37]. For example, ATF4, ATF6, and XBP-1 are typical regulated proteins involved in ER stress-induced apoptosis [38]. Surprisingly, in our study, we found that ATF4 played a leading role in the DAS triggered Noxa-dependent intrinsic apoptosis and CHOP-DR5 dependent extrinsic apoptosis, which coordinately activated the downstream caspase family (caspase3/7/9/8) and executed cell death.

Our results emphasized the vital role of p-eIF2*α*-ATF4 axis in apoptosis induced by DAS. However, it remains unclear how p-eIF2*α* is activated in the event. Previous studies indicated that PERK and GCN2 (general control nonderepressible 2) are key proteins responsible for eIF2*α* phosphorylation and activation [39, 40]. In addition, there is a hint that the heat shock protein 90 (HSP90) was a direct target of DAS [13], which might regulate the stability and function of PERK [41]. It is possible that DAS may promote the stabilization of PERK and phosphorylation of eIF2*α* in ER stress-related apoptosis. Moreover, DAS is a potent anti-cancer agent by regulating a variety of biological processes, and further studies are still needed to explore and identify crucial direct targets of DAS, as well as novel biological effects.

**Figure 9:**
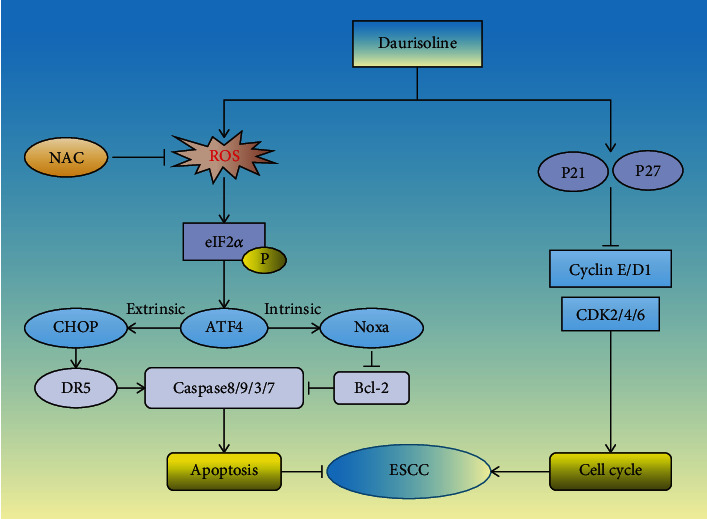
Working model of daurisoline inhibited the ESCC by inducing G1 cell cycle arrest and apoptosis.

In summary, we did a comprehensive study on the anti-cancer effect and mechanism of DAS on ESCC both *in vitro* and *in vivo* for the first time (Figure 9). The study provided critical insights into the molecular mechanism of DAS in ESCC therapy, which will shed new light on the research of anti-ESCC drugs through the discovery and identification of Chinese herbal ingredients.

## Figures and Tables

**Table 1:** Primers of genes for Q-PCR.

Gene	Forward primers (5′-3′)	Reverse primers (5′-3′)
*β*-Actin	CCCTGGAGAAGAGCTACGAG	TCCATGCCCAGGAAGGAAG
Noxa	ACCAAGCCGGATTTGCGATT	ACTTGCACTTGTTCCTCGTGG
DR5	CCAGCAAATGAAGGTGATCC	GCACCAAGTCTGCAAAGTCA
CHOP	AGCCAAAATCAGAGCTGGAA	TGGATCAGTCTGGAAAAGCA
ATF4	ATGACCGAAATGAGCTTCCTG	GCTGGAGAACCCATGAGGT

**Table 2:** siRNA sequences for target genes.

siRNA	Sequence (5′-3′)
Si-control	UUCUCCGAACGUGUCACGU
Si-p21#1	GACCAUGUGGACCUGUCAC
Si-p21#2	AAUGGCGGGCUGCAUCCAGGA
Si-p27#1	CCGACGAUUCUUCUACUCA
Si-p27#2	CCGACGAUUCUUCUACUCA
Si-Noxa#1	GGUGCACGUUUCAUCAAUUUG
Si-Noxa#2	CCGGCAGAAACUUCUGAAU
Si-DR5#1	AAGACCCUUGUGCUCGUUGUC
Si-DR5#2	CAGCCGUAGUCUUGAUUGU
Si-CHOP	GCCUGGUAUGAGGACCUGC
Si-ATF4#1	CCUCACUGGCGAGUGUAAA
Si-ATF4#2	GCCUAGGUCUCUUAGAUGA
Si-eIF2*α*#1	CGUAUCCGUUCUAUCAACAAACUCA
Si-eIF2*α*#2	AGCAGAUAUUGAAGUGGCUUGUUAU

## Data Availability

The original contributions presented in the study are included in the manuscript/supplementary material. Further inquiries can be directed to the corresponding authors.

## Supplementary Materials

Supplementary Materials Figure Supplement 1: (a) mouse body weight was recorded every other day during the whole experiment. (b) Hematoxylin and eosin (H&E) staining of liver and kidney were collected from the mice of the treatment and the control groups. (c) Serum of GGT, ALT, AST, CREA, and UA levels in DAS-treated mice compared with the control mice.
