# Supplementary material for: Daurisoline Inhibits ESCC by Inducing G1 Cell Cycle Arrest and Activating ER Stress to Trigger Noxa-Dependent Intrinsic and CHOP-DR5-Dependent Extrinsic Apoptosis via p-eIF2α-ATF4 Axis
Source: Oxid Med Cell Longev. 2022 Aug 4;2022:5382263. doi: 10.1155/2022/5382263 (PMC9371853; doi:10.1155/2022/5382263)
Supplement: Supplementary Materials — Figure Supplement 1: (a) mouse body weight was recorded every other day during the whole experiment. (b) Hematoxylin and eosin (H&E) staining of liver and kidney were collected from the mice of the treatment and the control groups. (c) Serum of GGT, ALT, AST, CREA, and UA levels in DAS-treated mice compared with the control mice. [file 5382263.f1.docx]

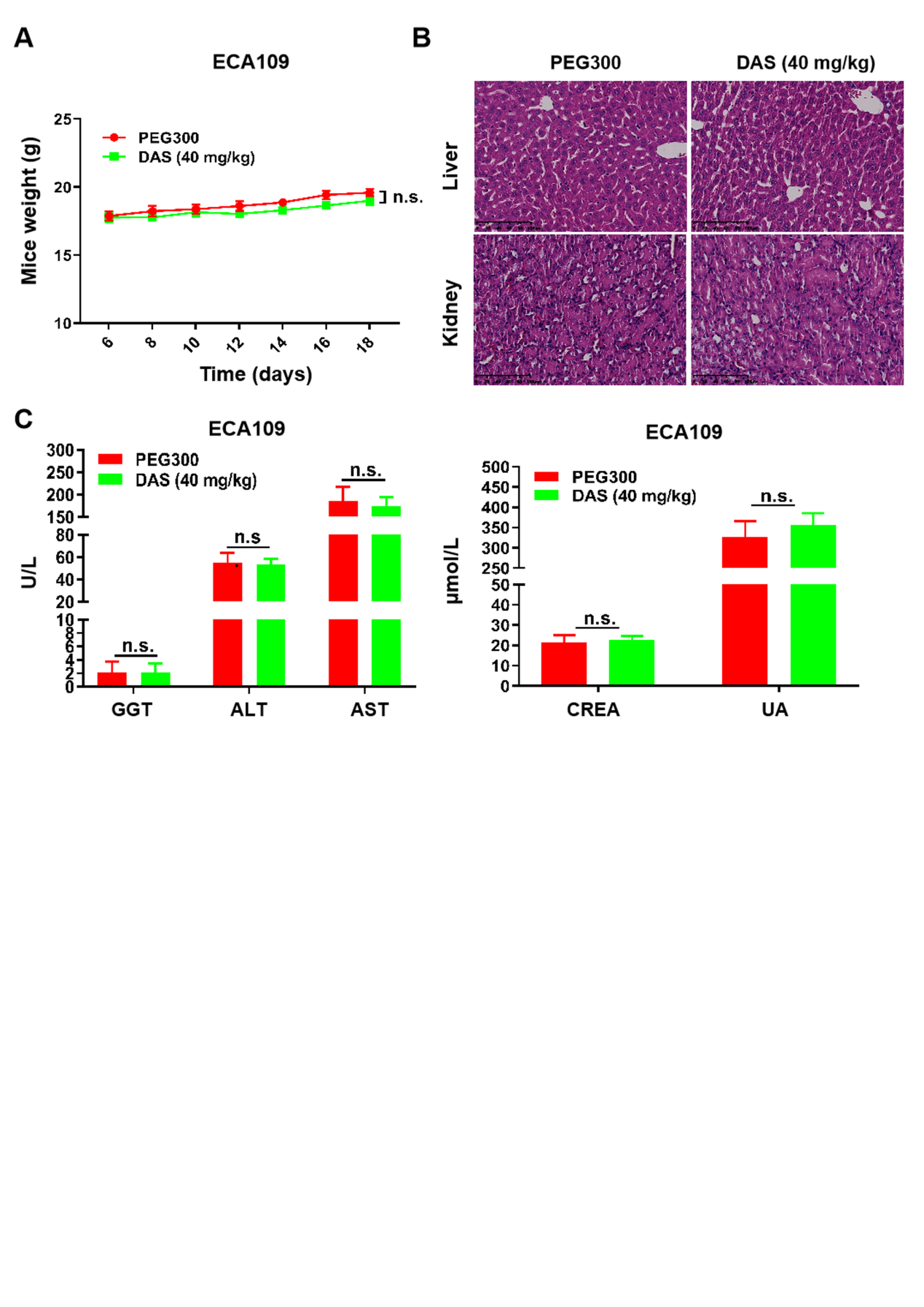


Figure Supplement 1 | (A) Mouse body weight was recorded every other day during the whole experiment. (B) Hematoxylin and eosin (H&E) staining of liver and kidney were collected from the mice of the treatment and the control groups. (C) Serum of GGT, ALT, AST, CREA, and UA levels in DAS-treated mice compared with the control mice.
